# An institutional investigation of false-positive signals in an automated blood culture system: evaluating clinical predictors and molecular etiologies

**DOI:** 10.1128/spectrum.02067-26

**Published:** 2026-09-23

**Authors:** Merve Ertürk Melez, Mustafa Altay Atalay, Pınar Sağiroğlu, Sıtkı Özgür Altop, Ayşe Nedret Koç

**Affiliations:** 1 Department of Medical Microbiology, Faculty of Medicine, Erciyes University 52958, Talas, Kayseri, Turkey; Taichung Veterans General Hospital, Taichung, Taiwan

**Keywords:** blood culture, false-positive signal, MALDI-TOF MS, PCR, next-generation sequencing

## Abstract

In bloodstream infections, initiating appropriate antimicrobial therapy promptly is crucial to reduce morbidity and mortality. Automated blood culture monitoring systems efficiently screen out negative specimens, allowing clinical laboratories to prioritize true-positive cultures. However, unconfirmed positive signals (false positives) present diagnostic challenges. This study aimed to perform an institutional evaluation of the false-positivity rate within the BD BACTEC FX system, assess pre-analytic and clinical variables, and investigate the potential presence of bacterial and fungal nucleic acids using MALDI-TOF MS, polymerase chain reaction, and next-generation sequencing (NGS). Over a 15-month period, 80 false-positive automated blood culture (ABC) bottles (positive growth signal but no growth on routine subculture) and 30 negative-signal controls were evaluated. Direct identification was attempted using MALDI-TOF MS (BD MALDI Biotyper Sirius, Bruker Daltonics). Bacterial and fungal DNA presence was investigated via amplification of bacterial 16S rRNA, fungal 28S rRNA, and ITS gene regions, followed by NGS targeting amplicons. Clinical parameters, time-to-positivity (TTP), and blood gas parameters were recorded. The institutional false-positivity rate was 0.2% (80/41,073 bottles), fully aligning with manufacturer specifications and published benchmarks. Bacterial DNA (16S rRNA) was not detected in any false-positive or negative control bottle. Fungal DNA (28S rRNA D1/D2 region) was amplified in 9 of 80 false-positive samples (11.25%); however, ITS region amplification was negative across all samples. Sequence analysis identified diverse environmental mold taxa (*Aspergillus melleus*, *Alternaria infectoria*, *Amorphotheca resinae*, *Alternaria conjuncta*, *Eremomyces bilateralis*, *Pseudomicrostroma glucosiphilum*, and *Aspergillus steynii*) in low relative abundance. No statistically significant differences were observed between fungal DNA-positive and negative groups regarding age, WBC count, or CRP levels (*P* > 0.05). All direct MALDI-TOF MS scores remained below 1.70, consistent with the absence of intact microbial protein biomass. The median TTP for the fungal DNA-positive false-positive bottles was 68.0 h, with several bottles taking up to 109 h to emit a signal. The BD BACTEC FX system demonstrated high operational specificity, with an institutional false-positivity rate of 0.2%. Comprehensive molecular analysis confirmed that false-positive signals did not conceal missed true-positive clinical pathogens. The sporadic identification of disparate fungal DNA in a minor subset of vials represents clinically insignificant environmental background contamination. Extending incubation periods of routine blood culture bottles is not recommended, as standard bottles are not formulated for mold recovery. In cases where mold infection is clinically suspected, direct subculturing onto dedicated fungal media or ordering dedicated fungal diagnostic testing is advised.

**IMPORTANCE:**

False-positive blood culture signals present a significant clinical and economic challenge, often leading to unnecessary antimicrobial treatment, extended hospital stays, and increased laboratory workloads. In this study, we investigated the underlying molecular etiology of these unexplained false-positive signals specifically occurring within the widely used BD FX automated blood culture system (BD Diagnostic Systems, USA). We believe this article is highly relevant to the readership of Microbiology Spectrum because it provides concrete molecular evidence of hidden fungal DNA contamination within commercial blood culture components. Since the BD BACTEC system is a global standard in clinical diagnostics, our findings offer highly actionable insights for clinical microbiologists worldwide to distinguish true clinical anomalies from technical artifacts, thereby reducing laboratory-driven diagnostic errors and improving antimicrobial stewardship.

## INTRODUCTION

Sepsis is a life-threatening clinical condition caused by a dysregulated host response to an infection, leading to organ dysfunction or even death. Globally, it is estimated that sepsis causes 11 million deaths annually, accounting for approximately 19.7% of all global deaths. Prolonged life expectancy, an increase in the number of immunosuppressed patients, and the widespread use of invasive procedures in intensive care units (ICUs) are among the main reasons why sepsis is increasingly becoming a public health problem (1).

Blood culture is still considered the “gold standard” method in the diagnosis of sepsis as it allows for the isolation of the causative microorganism and the performance of antimicrobial susceptibility testing. However, conventional blood culture methods can require a long period, such as 24–72 h, to obtain positive results and identify the pathogen. This delay leads clinicians to broad-spectrum empirical therapies, which in turn causes prolonged hospital stays, increased costs, and the development of antimicrobial resistance (2, 3).

Today, there is an increasing interest in rapid methods in clinical microbiology laboratories to fill this diagnostic gap. Molecular methods offer advantages such as rapid results and the detection of fastidious or unculturable microorganisms. However, these methods also come with their own challenges. The fact that blood culture bottles yield a positive signal in the automated system without containing viable microorganisms due to various clinical and technical reasons, such as high leukocyte count, excessive blood volume, or acidosis, is a significant problem termed “false positivity.” This situation leads to unnecessary antibiotic treatments, prolonged hospitalizations, and increased costs (4–7).

The primary objective of this study was to conduct a comprehensive institutional analysis of false-positive blood culture signals occurring within the BD BACTEC FX platform at a tertiary academic hospital. We evaluated pre-analytic and clinical predictors (including leukocyte counts, CRP, blood gases, and time-to-positivity [TTP]) and employed a multi-target molecular panel (bacterial 16S rRNA, fungal 28S rRNA, and ITS regions) and MALDI-TOF MS to determine whether unconfirmed signals represented missed true-positive infections or non-viable background nucleic acid traces.

## MATERIALS AND METHODS

### Study population and sample collection

This prospective study was selected from a total of 41,073 blood culture samples sent from ICUs, wards, and outpatient clinics to the Bacteriology Unit of Erciyes University Faculty of Medicine Central Laboratory between April 2022 and June 2023. The study included 80 false-positive blood culture bottles that gave a positive growth signal in the BD BACTEC FX Blood Culture System automated blood culture device but in which no microorganism could be detected by routine microbiological cultures (5% sheep blood agar, MacConkey agar, chocolate agar; 5–7 days incubation at 37°C) and direct Gram staining. In addition, 30 blood culture bottles that gave a negative signal at the end of 5 days of incubation in the automated blood culture device were included in the study as a control group. All bottles were stored at +4°C until the study. Blood culture bottles inoculated with *Staphylococcus aureus* ATCC 25923, *Klebsiella pneumoniae* ATCC 13883, and *Candida albicans* ATCC 90028, and one blood culture bottle each containing a gram-positive bacterium, a gram-negative bacterium, and *Candida* spp. whose growth was detected in routine culture, were used as positive controls. An uninoculated blood culture bottle was included as a negative control.

### Microorganism identification by MALDI-TOF MS

A modified method described in the literature was used for direct agent identification with the MALDI Biotyper Sirius System (Bruker Daltonics GmbH & Co. KG, Bremen, Germany) from 110 blood culture bottles (80 false positives + 30 negatives) included in the study. In this method, 1 mL of blood culture sample was transferred to a 1.5 mL sterile microcentrifuge tube, 100 µL of 10% Tween 80 solution was added, vortexed, and incubated for 5 min at room temperature. The mixture was centrifuged at 13,300 rpm for 6 min, and the supernatant was discarded. The pellet was purified by washing three times with 1 mL of distilled water. The sample taken from the obtained pellet was smeared as a thin layer on the target spot and left to air-dry. After drying, 1 µL of 70% formic acid and then 1 µL of α-Cyano-4-hydroxycinnamic acid matrix solution were added and dried at room temperature. The prepared spots were loaded into the MALDI Biotyper Sirius device, and results with an identification score above 1.70 were accepted.

### Molecular analysis steps

#### DNA isolation from samples

Genomic DNA isolation from a total of 110 blood samples yielding 80 false-positive and 30 negative signals, as well as from control bottles, was performed using the SuPure Blood DNA Isolation Kit (catalog no.: NA02R201, Ankara, Türkiye) according to the manufacturer’s instructions. The amount of isolated DNA was determined fluorometrically with Qubit 3.0 (Thermo Fisher Scientific, USA). The DNA isolation protocol included specific lysis, precipitation, and column washing steps as defined in the methodology.

#### Polymerase chain reaction amplification

- Amplification of fungal 28S rRNA D1/D2 regions: amplified using F63-F and LR3-R primer sequences (expected amplicon size: 600–650 bp).
- Amplification of fungal ITS regions: amplified using ITS1 and ITS4 primer sequences (expected amplicon size: 550–820 bp).
- Amplification of bacterial 16S rRNA V3–V4 region: amplified using 341F and 805R primer sequences (expected amplicon size: 464 bp).

#### Evaluation of polymerase chain reaction products

Polymerase chain reaction (PCR) products were evaluated by electrophoresis on a 1.5% agarose gel and visualized under UV light.

#### Library preparation and next-generation sequencing

PCR amplicons were purified using the HighPrep PCR Clean-up System. Library preparation was performed with the Nextera XT DNA Library Prep Kit, and sequencing was executed on the Illumina iSeq100 platform as paired-end 2 × 150 bp reads. Negative extraction controls were included to monitor for background DNA contamination.

#### Bioinformatics analysis of raw data

Raw reads were quality-controlled using FASTQC, and adapter regions were trimmed. Taxonomic classification was grouped into operational taxonomic units via the Kraken2 metagenomic system using the RefSeq 2022.02 database.

#### Controlled DNA spiking experiment

Following the molecular screening of the clinical cohort—where pan-bacterial 16S rRNA PCR was uniformly negative across all 80 false-positive bottles, whereas pan-fungal 28S rRNA D1/D2 PCR yielded positive amplifications in 11.25% (9/80) of the samples—a mechanistic spiking assay was sequentially designed. To evaluate system response to nucleic acids and assess whether cell-free fungal DNA could independently trigger the automated optical CO_2_ sensor, 15 standard blood culture vials (*n* = 3 per gradient: 0, 10, 100, 1,000, and 10,000 fungal genome copies/vial) obtained from active laboratory inventory lots were spiked with purified *Aspergillus* genomic DNA alongside sterile human plasma and monitored in the BD BACTEC FX system for 120 h. For this assay, genomic DNA was extracted in-house from reference *Aspergillus* isolates cultured under strict sterile conditions using the SuPure Blood DNA Isolation Kit with an additional mechanical and enzymatic fungal pre-lysis step. DNA concentration was precisely quantified fluorometrically with the Qubit 3.0 fluorometer (Thermo Fisher Scientific, USA), and nucleic acid purity was verified spectrophotometrically using a NanoDrop spectrophotometer, ensuring an A260/A280 absorbance ratio maintained between 1.80 and 1.95. Target genome copy numbers were calculated based on established fungal genome mass estimates, and serial working dilutions were prepared in molecular-grade, nuclease-free water.

### Statistical analysis

Descriptive statistics were presented as median (IQR) or mean ± SD. Since most variables did not exhibit a normal distribution (Shapiro-Wilk test), the non-parametric Mann–Whitney *U* test was used for comparisons between groups. A *P* value <0.05 was considered statistically significant. The rank-biserial *r*, Hodges-Lehmann estimate, and bootstrap 95% confidence intervals were also reported.

## RESULTS

### General study statistics

During the study period, a total of 41,073 blood culture bottles reached the Central Laboratory. Of these bottles, 8,312 (20.2%) gave a positive signal in the automated system, while 32,761 (79.8%) remained negative. Growth was detected in 8,232 (99%) of the 8,312 bottles giving a positive signal as a result of routine microbiological cultures. The remaining 80 blood culture bottles (1%) were evaluated as false-positive. The false-positivity rate among all bottles was determined to be 0.2%.

### MALDI-TOF MS results

With the MALDI Biotyper Sirius System, in direct identification attempts from a total of 110 blood culture samples (80 false-positive and 30 negative signals), the identification score of none of the samples exceeded 1.70. Therefore, microorganism identification directly from blood culture with MALDI-TOF MS was not successful in this study. Positive control bottles yielded scores above 1.70 and were identified correctly.

### Molecular analysis findings

As a result of the PCR analyses performed, no positivity for the bacterial (16S rRNA) or fungal ITS region was detected in any of the total 110 samples, including both false-positive and negative samples. However, a positive PCR product for the fungal 28S rRNA D1/D2 region was obtained in 9 (11.25%) of the 80 false-positive bottles. In the 30 negative-signal bottles, microorganism DNA was not detected by molecular methods. Positive controls generated compatible PCR products as expected, while uninoculated negative controls yielded no product. In the controlled spiking experiment (*n* = 15), blood culture bottles spiked with 10–100 genome copies/vial of purified Aspergillus DNA did not flag positive during 120 h of incubation. Vials spiked with extremely high non-physiological concentrations (≥1,000 copies) exhibited late metabolic background fluctuations. These results confirm that naked fungal nucleic acids do not directly modulate or trigger colorimetric CO_2_ sensors under normal physiological conditions.

In the nine samples subjected to next-generation sequencing (NGS) analysis, taxonomic distributions were examined at the species level. The obtained results reveal the fungal species found in the highest relative abundance in each sample (Table 1).

**TABLE 1:** Patient information of positive fungal PCR samples, species detected with highest relative abundance, diagnoses, and infection parameters^*a*^

Patient no.	Age	Gender	Ward	Microorganism	Diagnosis	WBC (4.8–10.7 × 10^3^/µL)	CRP (0–5 mg/L)	TTP (h)
14	13	M	PNS	*Aspergillus melleus*	Epilepsy	10.77	30.37	31
16	1	F	PHOS	*Amorphotheca resinae*	Adrenal gland malignant neoplasm	6.66	2.21	91
17	7	M	PHOS	*Alternaria conjuncta*	Brain malignant neoplasm	14.43	61.5	68
20	7	F	PHOS	*Eremomyces bilateralis*	Ewing sarcoma	0.5	104.74	86
23	40	F	IDS	*Pseudomicrostroma glucosiphilum*	Diabetes mellitus	11.75	26.41	109
33	1	F	NICU	*Aspergillus melleus*	Acute upper respiratory tract infection	11.92	43.9	108
34	73	M	GSICU	*Alternaria infectoria*	Pancreatic cancer	6.67	26.48	66
53	3	M	PHOS	*Aspergillus steynii*	Neuroblastoma	3.41	58.11	51
80	74	M	BNSICU	*Alternaria infectoria*	Intracranial hemorrhage	26.2	197	21

^*a*^ M, male; F, female; PNS, pediatric neurology service; PHOS, pediatric hematology oncology service; NICU, neonatal intensive care unit; GSICU, general surgery intensive care unit; BNSICU, brain nerve surgery intensive care unit; IDS, infectious diseases service.

### Patient and clinical data

The mean age of the 80 false-positive patients examined was 34.4; 50 were male and 30 were female. The mean leukocyte count of this group was 8,585/µL, and the mean CRP value was 60.68 mg/L. The mean age of the nine patients in whom fungal DNA was detected was 24.3 (five males and four females). Their mean leukocyte count was 10,256/µL, and the mean CRP value was 61.19 mg/L. According to patient epicrisis reports, fungal infection was not considered in any of the patients, and antifungal treatment was not initiated. Acidosis was detected in 3 of the 14 patients for whom blood gas analysis was requested (hypercapnia was also present in one patient). However, blood gas analysis had been requested for only two of the nine patients in whom the presence of microorganisms was demonstrated via the NGS method. An examination of the test results for these two patients revealed no findings of hypercapnia or acidosis.

Analysis of the TTP values for the fungal DNA-positive bottles (*n* = 9) yielded a median TTP of 68.0 h (IQR: 40.0; range: 21–109 h). When fungal DNA-positive (*n* = 9) and negative (*n* = 71) groups were compared, no statistically significant difference was found in terms of age, WBC, and CRP (Mann–Whitney *U*; age, *P* = 0.164; WBC, *P* = 0.183; CRP, *P* = 0.518). Effect sizes were small, and Hodges–Lehmann median differences and bootstrap 95% CIs included zero; hence, no clinically/statistically reliable difference was detected between the groups (Table 2).

**TABLE 2:** Mann–Whitney *U* test results for fungal DNA-positive and negative groups

Variable	Positive (*n* = 9), median (IQR)	Negative (*n* = 71), median (IQR)	*U*	*P* value
Age	7.0 (37.0)	20.0 (52.5)	227	0.164
WBC	10.77 (5.26)	6.0 (7.32)	391	0.183
CRP	43.9 (35.02)	28.86 (81.54)	353	0.518

## DISCUSSION

### Evaluation of general positivity and false-positivity rates

Continuous-monitoring automated blood culture systems represent a cornerstone of modern diagnostic microbiology. Given that a single blood culture set possesses a sensitivity of approximately 70%–80% for bacteremia detection, automated platforms play a vital role in screening out negative specimens (~80%), thereby allowing diagnostic laboratories to rapidly prioritize true-positive cultures (8, 9). Approximately 35% of blood cultures in patients with bloodstream infections are found to be positive. However, only 10% of all blood culture bottles arriving at laboratories are positive (10). In our study, 20.2% of the bottles gave a positive growth signal, and the culture positivity rate was found to be 20%. The low culture positivity rates can be explained by reasons such as empirical antibiotic therapy started before sampling, improper sampling, slow-growing pathogens, or insufficient blood volume (3, 11). False positivity in automated blood culture systems is a significant issue leading to unnecessary treatments and increased costs. In this study, the false-positivity rate of 0.2% in the BD BACTEC FX system is consistent with similar rates reported in the literature, confirming the high specificity and reliable performance of the system (4, 12–14).

### Other possible causes of false-positive signals

Several non-microbial factors can trigger false-positive signals in automated blood culture systems, including high leukocyte counts, patient acidosis, hypercapnia, bottle overfilling, and fastidious microorganisms (4, 5, 7). In our cohort, 25% of patients with false-positive signals had elevated leukocyte levels. Although systemic metabolic disturbances such as acidosis (*n* = 3) and hypercapnia (*n* = 1) were observed in a minor subset of patients tested for blood gases, none of the nine fungal DNA-positive patients presented with hypercapnia or acidosis. This indicates that host metabolic derangements were unlikely to be the primary cause of false positivity in the fungal DNA-positive subset. Furthermore, the ward distribution of the fungal DNA-positive cases showed no specific clustering (ranging from pediatric hematology-oncology and pediatric neurology to intensive care units and adult services).

### TTP

TTP serves as an essential parameter in distinguishing true bloodstream infections from contaminants or false signals; cultures that signal very late typically reflect non-viable cell debris, low inoculum artifacts, or technical false positives (15). In our study, the median TTP for the fungal DNA-positive false-positive bottles was 68.0 h, with several bottles taking up to 109 h to emit a signal.

This prolonged TTP strongly supports our hypothesis that the positive growth signals were not caused by actively replicating clinical fungal pathogens. Instead, it suggests a slow, background physicochemical interaction or fluorescence sensor alteration triggered by non-viable, highly fragmented fungal DNA components and potential manufacturing-derived chemical residues present within the culture bottles.

### Molecular research in false-positive blood culture bottles

In a study by Wellinghausen et al. (2), the presence of bacteria in the blood was investigated using PCR and sequence analysis targeting the 16S rRNA gene region. Out of 288 blood culture-negative samples, 41 yielded positive PCR results. Statistical analysis indicated that the false-positive rate of the test remained within acceptable limits. While molecular diagnostic methods entail significantly higher costs compared to standard blood culture protocols, they offer distinct advantages. PCR testing provides a shorter time-to-result and exhibits higher diagnostic sensitivity, particularly through the detection of non-viable pathogens. However, to properly manage PCR-positive/culture-negative cases (potential false positives), it is recommended to integrate PCR results with ancillary microbiological cultures and patient clinical data (2).

Other researchers have reported differing results. Marlowe et al. were unable to detect bacterial or fungal presence in false-positive blood culture bottles using DNA probe analysis (16). Similarly, Daxboeck et al. found no positive results in false-positive bottles using 18S rDNA and 16S rDNA amplification (17). Conversely, Petit et al. identified positivity in 5 out of 181 false-positive bottles by employing molecular approaches, including scanning electron microscopy and NGS (7). In our present study, fungal presence was detected via PCR in 11.25% of the blood culture bottles that emitted false-positive signals.

### MALDI-TOF MS findings interpretation

MALDI-TOF MS requires a specific threshold of protein density for accurate microbial identification (18). One of the most significant limitations in using the MALDI-TOF MS method for mold identification is the lack of extensive, commercially available databases (19). The primary finding of our molecular investigation was that false-positive bottles did not conceal missed true-positive clinical pathogens. Bacterial 16S rRNA PCR was negative across all false-positive specimens, and direct MALDI-TOF MS scores remained below 1.70. As noted in clinical microbiology practice, the inability of direct MALDI-TOF MS to yield identification scores is most straightforwardly explained by the absence of intact, viable microbial protein biomass in these bottles.

### Molecular findings interpretation

Sequencing of the fungal 28S rRNA D1/D2 region and the ITS regions allows for the molecular identification of most fungal species (20). Fungal DNA targeting the 28S rRNA D1/D2 region was detected in a minor subset (11.25%, 9/80) of false-positive bottles, whereas ITS region PCR was uniformly negative. The failure to amplify the ITS barcode region suggests that the detected DNA represents highly fragmented, non-viable nucleic acid traces.

Most of the fungal species detected by NGS, particularly *Amorphotheca resinae*, *Alternaria conjuncta* (new name *A. scrophulariae*), *Eremomyces bilateralis*, *Pseudomicrostroma glucosiphilum*, and *Aspergillus steynii*, are considered widespread environmental contaminants (21–23). There are no publications in the literature reporting that these species cause fungemia in humans. Although species such as *Aspergillus melleus* and *Alternaria infectoria* can cause clinical cases like onychomycosis or sinusitis, there is no evidence of them causing fungemia (24–28). These fungal species identified by NGS represent common environmental molds. In our clinical cohort, pan-bacterial 16S rRNA PCR was uniformly negative across all 80 false-positive bottles, whereas pan-fungal 28S rRNA D1/D2 PCR yielded positive amplifications in 11.25% (9/80) of the samples. Consequently, the mechanistic spiking experiment was sequentially designed and purposefully tailored to test the specific hypothesis of whether free or naked fungal DNA could independently trigger the automated optical CO_2_ sensor in the BD BACTEC FX system. In the spiking experiment, the rare signals observed at extremely high artificial concentrations (≥1,000 copies) were accompanied by minor metabolic background fluctuations rather than direct sensor modulation by DNA. It can be concluded that the low levels of fungal DNA detected in a small subset (11.25%) of the bottles yielding false-positive results represent incidental environmental nucleic acid fragments or trace amounts of spores introduced into the system during production or sampling, rather than the primary mechanism triggering the device. Importantly, our findings indicate that free fungal DNA does not directly trigger the colorimetric CO_2_ sensor of blood culture vials. As previously demonstrated by Qian et al. (12), nucleic acids do not interact with CO_2_ monitoring sensors. Furthermore, clinical record review confirmed that none of the nine patients were clinically diagnosed with fungal sepsis or administered antifungal therapy. Pre-analytic factors such as elevated host cell metabolic activity (leukocytosis), bottle blood overfilling, or transient hypercapnia remain the primary physiological drivers of unconfirmed growth signals. Trace mold DNA identified in a minor fraction of vials is an incidental background finding, likely introduced during collection or ambient laboratory processing.

### Importance of clinical correlation

The lack of statistically significant differences in inflammation markers, such as WBC and CRP, between groups with and without detected fungal DNA appears consistent with the absence of clinical infection. When the clinical data of nine patients in whom fungal DNA was detected by molecular methods were examined, it was seen that four of these patients were immunosuppressed due to a diagnosis of malignancy, and most had elevated leukocyte and CRP levels. This situation may indicate the presence of an ongoing infection or inflammatory process in these individuals. However, the fact that most of the fungal species detected by NGS are not known sepsis agents strengthens the idea that the main cause of the patients’ clinical picture may not be these detected DNA fragments, but rather a different pathogen that cannot be produced in routine culture or is located in another focus. Although the detection of fungal DNA is not evidence of active fungemia, further studies should investigate whether it may be an indirect indicator of increased environmental exposure or colonization in non-sterile areas such as mucosa in this susceptible patient group. Therefore, such molecular findings should be interpreted by carefully integrating them with the clinical picture, especially in immunosuppressed patients, in order to avoid inappropriate empirical antifungal treatments.

### Limitations

This study has several limitations. First, because the clinical false-positive specimens and baseline controls were collected dynamically across a 15-month operational timeframe, they represented multiple independent commercial manufacturing lots supplied to the central laboratory. Although this multi-lot longitudinal sampling reflects routine real-world performance, lot-matched, unopened sterile vials directly from the manufacturer were not available for parallel sequencing. Consequently, inter-lot chemical or environmental variations could theoretically introduce minor fluctuations, preventing definitive attribution of background DNA traces strictly to manufacturing processes versus sampling environments. Additionally, the culture bottles utilized in the controlled spiking experiments were obtained from active laboratory inventory lots at the time of testing and could not be retrospectively lot-matched to each individual clinical false-positive bottle collected over the 15-month study period, which could potentially introduce unmeasured inter-batch variations. Future prospective investigations evaluating manufacturer-supplied, unopened lot-matched controls in parallel with clinical vials are warranted. Second, because fungal DNA was detected in only 11.25% of false-positive bottles, the presence of nucleic acids cannot account for the majority of unconfirmed signals. Third, blood fill volumes could not be retrospectively evaluated because the blood culture bottles were discarded following institutional safety protocols upon expiration. Finally, routine laboratory evaluation of false-positive bottles relied on standard bacterial subculture media (5% sheep blood, MacConkey, and chocolate agar) incubated for 5–7 days at 37°C. Because dedicated fungal media and extended room-temperature incubation were not routinely applied at the time of initial processing, non-viable or fastidious environmental mold spores were not targeted for mycological culture recovery.

### Conclusion and recommendations

In conclusion, this institutional investigation demonstrates that the BD BACTEC FX automated blood culture system performs highly reliably, maintaining a low false-positivity rate of 0.2%. Comprehensive molecular analysis confirmed that false-positive signals did not conceal missed true-positive clinical pathogens. The sporadic identification of disparate fungal DNA in a minor subset of bottles (11.25%) represents clinically insignificant environmental background contamination. Extending incubation periods of routine blood culture bottles is not recommended, as standard bottles are not formulated for mold recovery. In cases where mold infection is clinically suspected, direct subculturing onto dedicated fungal media or ordering dedicated fungal diagnostic testing is advised. Clinical management of false-positive signals should rely on thorough patient evaluation and integration with commercial molecular panels to support antimicrobial stewardship.

## Data Availability

The data sets generated and analyzed during the current study are available from the corresponding author upon reasonable request.
